# Supplementary material for: An empirical study of drone medical logistics transportation in a multi-campus model of Chinese public hospitals: Real-world data-driven validation of timeliness and application effects
Source: PLoS One. 2026 Mar 20;21(3):e0345282. doi: 10.1371/journal.pone.0345282 (PMC13004373; doi:10.1371/journal.pone.0345282)
Supplement: S5 Code — (DOCX) [file pone.0345282.s005.docx]

import matplotlib.pyplot as plt

import numpy as np

# Set academic style with Times New Roman font

plt.rcParams['font.family'] = 'Times New Roman'

plt.rcParams['mathtext.fontset'] = 'stix' # For mathematical symbols

# Data for Chart 1: Comparison at midnight (most uncongested period)

methods = ['Drone', 'Baidu Map\n(Road)', 'Gaode Map\n(Road)', 'Tencent Map\n(Road)']

distances = [5.95, 6.2, 6.2, 6.2]

distance_errors = [0.03, 0, 0, 0]

unit_times = [1.64, 2.06, 2.01, 2.03]

unit_time_errors = [0.14, 0.12, 0.12, 0.09]

# Create Chart 1

fig1, ax1 = plt.subplots(figsize=(10, 6))

# Unit time comparison bar chart

bars = ax1.bar(methods, unit_times, yerr=unit_time_errors, capsize=8,

color=['#2E86AB', '#A23B72', '#A23B72', '#A23B72'],

alpha=0.8, width=0.6)

ax1.set_ylabel('Unit Time (min/km)', fontsize=12)

ax1.set_title('Comparison of Drone and Road Traffic Efficiency\n(Midnight - Most Uncongested Period)', fontsize=14, fontweight='bold')

ax1.grid(axis='y', alpha=0.3)

# Add values on bars

for i, (v, e) in enumerate(zip(unit_times, unit_time_errors)):

ax1.text(i, v + e + 0.05, f'{v}±{e}', ha='center', va='bottom', fontsize=10)

# Add distance information annotations with further adjusted position

for i, method in enumerate(methods):

if i == 0: # Drone

distance_info = f'Distance: {distances[i]}±{distance_errors[i]} km'

else: # Road traffic

distance_info = f'Distance: {distances[i]} km'

# Position text slightly lower to create more space

ax1.text(i, -0.25, distance_info, ha='center', va='top', fontsize=11,

style='italic')

# Adjust y-axis range to make room for bottom annotations

ax1.set_ylim(0, 2.5)

# Add data source with larger font and adjusted position

plt.figtext(0.5, 0.01, "Data source: 751 drone flights vs. 30-day continuous measurement by navigation apps",

ha="center", fontsize=11, style='italic')

plt.tight_layout()

plt.subplots_adjust(bottom=0.14) # Further increased bottom margin

# Save as high-resolution image

plt.savefig('Drone_vs_Road_Midnight.png', dpi=300, bbox_inches='tight',

facecolor='white', edgecolor='none')

plt.savefig('Drone_vs_Road_Midnight.pdf', bbox_inches='tight',

facecolor='white', edgecolor='none')

plt.show()

# Data for Chart 2: All-day comparison

time_points = ['0:00', '8:00', '10:00', '12:00', '14:00', '16:00', '18:00', '20:00', '22:00']

road_unit_times = [2.03, 3.39, 4.12, 2.67, 2.61, 2.65, 3.22, 2.23, 2.09]

road_errors = [0.09, 0.12, 0.09, 0.10, 0.12, 0.18, 0.12, 0.14, 0.90]

drone_unit_time = 1.64

drone_error = 0.13

# Create Chart 2

fig2, ax2 = plt.subplots(figsize=(12, 7))

# Plot road transport time curve

(line,) = ax2.plot(time_points, road_unit_times, 'o-', linewidth=2, markersize=8,

color='#A23B72', label='Road Traffic Unit Time (Tencent Map)')

ax2.errorbar(time_points, road_unit_times, yerr=road_errors, fmt='o', color='#A23B72',

capsize=5, capthick=2, alpha=0.7)

# Plot drone time horizontal line

ax2.axhline(y=drone_unit_time, color='#2E86AB', linestyle='--', linewidth=2,

label=f'Drone Unit Time ({drone_unit_time}±{drone_error} min/km)')

ax2.fill_between(time_points, drone_unit_time - drone_error, drone_unit_time + drone_error,

alpha=0.2, color='#2E86AB')

# Set chart properties

ax2.set_xlabel('Time of Day', fontsize=12)

ax2.set_ylabel('Unit Time (min/km)', fontsize=12)

ax2.set_title('All-day Comparison of Road Traffic and Drone Efficiency', fontsize=14, fontweight='bold')

ax2.legend(loc='upper left', framealpha=0.8)

ax2.grid(True, alpha=0.3)

# Set y-axis range to provide enough space for annotations

ax2.set_ylim(1.2, 5.0)

# Mark key time points with optimized positions to avoid overlap

# Outpatient peak (10:00)

ax2.annotate('Outpatient\nPeak',

xy=(2, road_unit_times[2]),

xytext=(2.5, 4.5),

arrowprops=dict(arrowstyle='->', color='red', lw=1.5),

bbox=dict(boxstyle="round,pad=0.3", facecolor="mistyrose", alpha=0.8, edgecolor='red'),

ha='center', fontweight='bold', fontsize=10)

# Morning peak (8:00)

ax2.annotate('Morning\nPeak',

xy=(1, road_unit_times[1]),

xytext=(0.5, 3.7),

arrowprops=dict(arrowstyle='->', color='orange', lw=1.5),

bbox=dict(boxstyle="round,pad=0.3", facecolor="lightyellow", alpha=0.8, edgecolor='orange'),

ha='center', fontweight='bold', fontsize=10)

# Evening peak (18:00)

ax2.annotate('Evening\nPeak',

xy=(6, road_unit_times[6]),

xytext=(7, 3.7),

arrowprops=dict(arrowstyle='->', color='orange', lw=1.5),

bbox=dict(boxstyle="round,pad=0.3", facecolor="lightyellow", alpha=0.8, edgecolor='orange'),

ha='center', fontweight='bold', fontsize=10)

# Most uncongested (0:00) - moved upward

ax2.annotate('Most\nUncongested',

xy=(0, road_unit_times[0]),

xytext=(0.9, 2.2), # Increased from 1.8 to 2.2

arrowprops=dict(arrowstyle='->', color='green', lw=1.5),

bbox=dict(boxstyle="round,pad=0.3", facecolor="honeydew", alpha=0.8, edgecolor='green'),

ha='center', fontweight='bold', fontsize=10)

# Add efficiency analysis box

time_savings = [15.79, 2.83] # Max and min time savings in minutes

textstr = '\n'.join([

'Efficiency Analysis:',

f'Max saving: {time_savings[0]} min',

f'Min saving: {time_savings[1]} min'

])

props = dict(boxstyle='round', facecolor='lightblue', alpha=0.7, edgecolor='navy')

ax2.text(0.75, 0.85, textstr, transform=ax2.transAxes, fontsize=10,

verticalalignment='top', bbox=props)

# Add data source with larger font

plt.figtext(0.5, 0.01, "Data source: 751 drone flights vs. Tencent Map 30-day continuous measurement",

ha="center", fontsize=11, style='italic')

plt.tight_layout()

plt.subplots_adjust(bottom=0.1)

# Save as high-resolution image

plt.savefig('All-day_Efficiency_Comparison.png', dpi=300, bbox_inches='tight',

facecolor='white', edgecolor='none')

plt.savefig('All-day_Efficiency_Comparison.pdf', bbox_inches='tight',

facecolor='white', edgecolor='none')

plt.show()
