# Supplementary material for: An empirical study of drone medical logistics transportation in a multi-campus model of Chinese public hospitals: Real-world data-driven validation of timeliness and application effects
Source: PLoS One. 2026 Mar 20;21(3):e0345282. doi: 10.1371/journal.pone.0345282 (PMC13004373; doi:10.1371/journal.pone.0345282)
Supplement: Attachment — Submitted filename: Response to Reviewers.docx [file pone.0345282.s007.docx]

**Point-by-point response to editors and reviewers’ comments**

Dear Editors and Reviewers

Thank you for your valuable suggestions and for the reviewers' comments concerning our manuscript entitled“An empirical study of drone medical logistics transportation in a multi-campus model of Chinese public hospitals: real-world data-driven validation of timeliness and application effects”(ID:PONE-D-25-54130). These comments are all valuable and very helpful for revising and improving our manuscript, as well as the important guiding significance to our researches. We have studied comments carefully and have made correction which we hope meet with approval. Revised portion are marked in red in the manuscript. The main corrections in the manuscript and the responds to editors and reviewers’ comments are as following:

**Regarding the issues raised on January 14, 2026, point-by-point response to editors:**

1 & 2. Regarding the update to the author list and the request for an Authorship Change Form

Response: Thank you for your instructions. We have completed the two required actions regarding authorship for our manuscript (ID: PONE-D-25-54130):

Authorship Change Request Form: The completed forms have been uploaded to the submission system (file type ‘Other’).

Written Confirmation from All Authors: As requested, all co-author have sent their written consent statement directly to your editorial office(plosone@plos.org), confirming their agreement to the restored author list. The compilation of author consent emails was uploaded to file inventory.

For your reference, the corresponding email addresses for all authors are:

Xingbo Long (Corresponding Author): [723766496@qq.com]

Min Huang: [993557884@qq.com]

Xiankai Xie: [kellandx@qq.com(75502266@qq.com)]

Yanxin Wang: [1078439109@qq.com]

Xiaojiang Yu: [822797265@qq.com]

Wei Zhou: [10205242@qq.com]

Bo Hu: [975171797@qq.com]

Thank you again for your time and consideration. We believe our manuscript now fully complies with the journal's policies and are grateful for the opportunity to clarify these points.

**Regarding the issues raised on November 5, 2025, point-by-point response to editors:**

1. Please ensure that your manuscript meets PLOS ONE's style requirements, including those for file naming. The PLOS ONE style templates can be found at https://journals.plos.org/plosone/s/file?id=wjVg/PLOSOne_formatting_sample_main_body.pdf and https://journals.plos.org/plosone/s/file?id=ba62/PLOSOne_formatting_sample_title_authors_affiliations.pdf

Response: We have thoroughly reformatted the entire manuscript in strict accordance with the formatting template provided by PLOS ONE. Specifically, we utilized the paper template from the link you provided and adjusted the font, line spacing, section headings, figure and table captions, and reference formatting. The revised main manuscript file has been named as requested: Revised Manuscript with Track Changes.docx.

2. In your Methods section, please include additional information about your dataset and ensure that you have included a statement specifying whether the collection and analysis method complied with the terms and conditions for the source of the data.

Response: In the “Data Sources and Collection” subsection of the “Materials and Methods” section, we have supplemented a detailed description: “The primary data for this study comprised drone flight data and ground road travel times. The former was directly exported from the project's drone management system, while the latter was obtained by measuring travel times between the two campuses using navigation software. Acquisition and Compliance Statement for Ground Road Traffic Time Benchmarks. To establish a benchmark for comparing delivery timelines with drone transportation, this study manually queried and recorded predicted road travel times under actual traffic conditions for routes between the same starting and ending campuses during the drone test period. This data was obtained by accessing the publicly available, free real-time route planning and navigation features provided by Baidu Maps, Amap, and Tencent Maps through their official mobile applications. These functions constitute standardized, free services offered to the public. We hereby confirm that this data acquisition method fully complies with the terms regarding personal, non-commercial, and reasonable use outlined in the platforms' publicly available Terms of Service. The collected data is exclusively used for comparative analysis within this academic research and has not been utilized for any commercial purposes or bulk scraping.” Compliance Statement: We confirm that the data acquisition and analysis methods employed in this study fully comply with relevant regulations.

3. Please note that PLOS One has specific guidelines on code sharing for submissions in which author-generated code underpins the findings in the manuscript. In these cases, we expect all author-generated code to be made available without restrictions upon publication of the work. Please review our guidelines at https://journals.plos.org/plosone/s/materials-and-software-sharing#loc-sharing-code and ensure that your code is shared in a way that follows best practice and facilitates reproducibility and reuse.

Response: This study employed custom-written code and utilized Python for data visualization. We have adhered to PLOS ONE's code-sharing mechanism to ensure unrestricted access and reuse. Additionally, we have included a “Data availability” statement and “Supporting information” at the end of the manuscript.

4. In your Methods section, please provide additional information regarding the permits you obtained for the work. Please ensure you have included the full name of the authority that approved the field site access and, if no permits were required, a brief statement explaining why.

Response: We have supplemented the following information in the “Research Design and Ethical Approval” subsection of the “Materials and Methods” section: “This study constitutes a retrospective systematic evaluation and analysis based on hospital operational data. It does not involve any clinical trials involving human or animal subjects, nor does it collect or utilize any personally identifiable information (including personal details of patients, medical staff, or other participants). All analyzed data pertains exclusively to project operational metrics, such as flight frequencies, transport durations, and cost data. Therefore, in accordance with the exemption provisions outlined in the World Medical Association's Declaration of Helsinki and China's Ethical Review Measures for Biomedical Research Involving Human Subjects, the Ethics Committee of Deyang People's Hospital has reviewed and confirmed that this study is exempt from ethical review (Ethics Review Number: 2024-03-004-K01). All data processing procedures in this study strictly adhere to the hospital's management regulations concerning data security and privacy protection.” Therefore, in accordance with relevant regulations, this study did not seek additional ethical committee approval.

5. Thank you for stating the following financial disclosure:

“This work was supported by National Institute of Hospital Administration [grant number 2025RWE023]; Sichuan Province Health Information Center [grant number 2021ZXKY06012]; Sichuan Province Health Information Society [grant number 2022051]; and Sichuan Provincial Hospital Association [grant number SCZB013].”

Please state what role the funders took in the study. If the funders had no role, please state: "The funders had no role in study design, data collection and analysis, decision to publish, or preparation of the manuscript."

If this statement is not correct you must amend it as needed.

Please include this amended Role of Funder statement in your cover letter; we will change the online submission form on your behalf.

Response: We acknowledge that the specific roles of the funding sources in this study are as follows: National Institute of Hospital Administration, Sichuan Province Health Information Center, Sichuan Province Health Information Society and Sichuan Provincial Hospital Association provided financial support for this study. Beyond this, the funding sources did not participate in the study design, data collection and analysis, publication decisions, or manuscript writing. Please assist in updating the funding declaration in the online submission form to: “This work was supported by National Institute of Hospital Administration [grant number 2025RWE023]; Sichuan Province Health Information Center [grant number 2021ZXKY06012]; Sichuan Province Health Information Society [grant number 2022051]; and Sichuan Provincial Hospital Association [grant number SCZB013]. The funders had no role in study design, data collection and analysis, decision to publish, or preparation of the manuscript.”

6. Thank you for stating the following in the Acknowledgments Section of your manuscript:“This work was supported by National Institute of Hospital Administration [grant number 2025RWE023]; Sichuan Province Health Information Center [grant number 2021ZXKY06012]; Sichuan Province Health Information Society [grant number 2022051]; and Sichuan Provincial Hospital Association [grant number SCZB013]”

We note that you have provided funding information that is not currently declared in your Funding Statement. However, funding information should not appear in the Acknowledgments section or other areas of your manuscript. We will only publish funding information present in the Funding Statement section of the online submission form.

Please remove any funding-related text from the manuscript and let us know how you would like to update your Funding Statement. Currently, your Funding Statement reads as follows:

“This work was supported by National Institute of Hospital Administration [grant number 2025RWE023]; Sichuan Province Health Information Center [grant number 2021ZXKY06012]; Sichuan Province Health Information Society [grant number 2022051]; and Sichuan Provincial Hospital Association [grant number SCZB013].”

Please include your amended statements within your cover letter; we will change the online submission form on your behalf.

Response: We have fully removed all references to the previously mentioned funding source and grant number from the manuscript as requested. We have included our amended statements within our cover letter. We hereby formally request that the “Funding Statement” in our online submission system be updated to: “This work was supported by National Institute of Hospital Administration [grant number 2025RWE023]; Sichuan Province Health Information Center [grant number 2021ZXKY06012]; Sichuan Province Health Information Society [grant number 2022051]; and Sichuan Provincial Hospital Association [grant number SCZB013].”

7. If the reviewer comments include a recommendation to cite specific previously published works, please review and evaluate these publications to determine whether they are relevant and should be cited. There is no requirement to cite these works unless the editor has indicated otherwise.

Response: We have carefully reviewed the specific literature cited in the reviewer's suggestions. After evaluation, two of the references pertain to robotics: one concerns the design and development of food delivery robots, while the other addresses robotic applications in corn cultivation. These topics differ from the medical logistics drone applications investigated in our study and thus were not included. We appreciate the reviewer's valuable suggestions.

**Point-by-point response to reviewers:**

**Reviewer #1:**

1. The abstract includes excessive numerical data and statistical details, which make it difficult to follow and obscure the main findings. The authors should summarize key performance outcomes concisely and focus on the broader implications of UAV-based logistics rather than listing every numerical result.

Response: We fully agree with your perspective. In the revised abstract, we have removed secondary numerical results. Simultaneously, we have strengthened the presentation of the study's primary conclusions and their broader implications for the field of medical logistics using drones, enhancing the abstract's readability and conciseness. Specific modifications can be found on page 1 of the manuscript.

2. Although the study claims to “pioneer” UAV feasibility evaluation for medical logistics, it lacks a clear explanation of what distinguishes this work from existing studies in medical UAV transport. The authors should also acknowledge methodological or operational limitations—such as regulatory, weather, or payload constraints—to make the abstract more balanced and realistic.

Response:

Novelty: In the “1. Introduction” section, we added subsection “1.2.2 Limitations of existing research and innovations of this study,” highlighting how this research differs from existing literature: This study systematically evaluates the timeliness and stability of drone medical logistics in a multi-hospital urban setting in China for the first time, utilizing real-world flight data from 750 missions. It innovatively incorporates comparisons of multiple navigation software, round-the-clock monitoring, and large-effect-size analysis, providing high-quality evidence for the practical application of drone medical logistics. Specific modifications are detailed on pages 5 to 6 of the manuscript.

Limitations: Although this study yielded valuable findings, certain limitations exist. In the “4. Discussion” section, we have added subsection “4.4.1 Limitations of this study” to candidly discuss the boundaries of this research, including but not limited to: the single-center study design limiting the generalizability of results, and the insufficient consideration of weather variation effects. Specific revisions can be found on pages 26 to 27 of the manuscript.

3. The introduction should clearly conclude with a distinct paragraph that highlights the novel contributions of your work.

Response: Indeed, the introduction should include a distinct paragraph summarizing the novel contributions of this research. We have added a separate section titled “1.3 Research Objectives and Questions” at the end of the introduction, clearly outlining the core issues addressed by this study and the anticipated primary academic or practical contributions. This provides readers with a clear research roadmap.

4. The literature review should benefit from more explorations of previous studies.

Response: In the “1. Introduction” section, we have expanded the “Literature Review” or ‘Background’ section. Not only have we increased the number of recent key references, but more importantly, we have strengthened the critical analysis of the existing research landscape. This allows for a more natural introduction to the necessity of this study. Please see the revision in “1.2 Literature Review” on page 4.

5. The discussion section needs to be expanded to more thoroughly analyze the results.

Response: We have substantially rewritten and expanded the Discussion section. The revised discussion:

- No longer merely reiterates results but delves into the underlying causes and mechanisms behind key findings.

- Provides detailed comparisons between our results and those from similar or dissimilar studies reported in the literature, explaining both similarities and differences.

- Explores specific implications of the findings for policymakers, hospital administrators, and technology developers.

Revisions can be found on pages 21–30.

6. The first paragraph of the conclusion should succinctly summarize the contributions of the study in past tense.

Response: We have rewritten the conclusion section. The first paragraph of the conclusion now summarizes the main work completed and core findings of this study in the past tense, clearly and concisely, directly addressing the research objectives. Please see page 30 for the revisions.

7. The second paragraph of the conclusion should provide clear and actionable future recommendations.

Response: In the Discussion section, we have transformed the second paragraph of the Conclusions into a forward-looking “Research Directions” and “Practical Application Recommendations” section. Here, we present specific, actionable suggestions. For example: Regarding follow-up research, we recommend exploring the integrated application of emerging technologies such as blockchain. For practical implementation, we recommend establishing multidisciplinary teams comprising clinicians, laboratory technicians, logistics management experts, aviation technicians, and information engineers to jointly advance the clinical translation and continuous optimization of drone-based medical logistics. Regarding policy, we suggest actively engaging with local airspace management authorities and health administration departments to participate in developing regional standards and industry regulations for drone medical logistics, thereby fostering a favorable policy environment for technology adoption. Please refer to pages 27–30 for the revised sections.

8. Some equations are not properly cited.

Response: To enhance the readability and academic rigor of this paper, we have restructured the entire manuscript. After careful consideration, we have removed all equation references from the previous version of the manuscript.

9. Visualizations from Table 3 would add good value to the manuscript.

Response: This is an excellent suggestion. We have added two figures (Figures 3 and 4) to provide visual representation, and this modification has significantly enhanced the clarity and impact of the results presentation. The revisions can be found on pages 14 and 15 of the manuscript.

**Reviewer #2:**

1. The introduction section should provide a clearer explanation of the aim and motivation of the study, as the current version is brief and lacks sufficient context. Additional references and detailed explanations should be incorporated to strengthen the introduction. The research objectives are not explicitly stated and should be clearly outlined to guide readers regarding the purpose and focus of the paper. The related work section could be enhanced by including more relevant and recent studies to provide a stronger foundation for the research.

Response: We expanded the literature review in the introduction section, incorporating two major parts: “1.2.1 Types and Application Scenarios of Medical Drone Transportation” and “1.2.2 Limitations of Existing Research and Innovations of This Study.” Key literature from the past five years on medical drone applications and smart hospital logistics has been systematically supplemented and referenced, strengthening the research foundation.

At the conclusion of the introduction, we introduced a new subsection titled “1.3 Research Objectives and Questions.” This section clearly articulates the study's purpose and motivation in bullet points, guiding readers to understand the paper's core objectives and central focus.

Please refer to pages 4-6 of the manuscript for the revisions.

2. The conclusion section could be improved by providing a more detailed summary of the findings and highlighting the key contributions of the study. While the paper is generally readable, minor grammatical issues should be revised to improve clarity and overall presentation. The reference list is limited and should be extended with additional relevant and recent works, and the formatting of references should be made consistent and professional.

Response: We have rewritten the Conclusions section. The first paragraph concisely summarizes the core research findings using the past tense. Please refer to page 30 of the manuscript for the revisions.

We have corrected minor grammatical and stylistic issues to enhance the clarity and readability of the manuscript. Strictly adhering to PLOS ONE formatting requirements, we have expanded the reference list to 43 entries, ensuring all entries are complete and uniformly formatted.

3. Figures and tables should be discussed in greater detail within the text to improve interpretation and context. All figures should be properly referred to in the text and briefly explained. Tables should have their captions positioned above the table, and the content should be explained in more detail for clarity. Each equation or algorithm should be fully explained, including a description of all parameters used.

Response: In the “Results” section of the main text, we have added dedicated descriptive and analytical paragraphs for each figure and table, explaining the core information they present and their supporting role in the research argumentation. The modified sections have been highlighted in red within the main text.

We have ensured that all table titles are positioned above the tables and are more descriptive in content; all figures and tables referenced in the main text are now explicitly cited.

4. The introduction could benefit from incorporating the following references to enhance depth and recency:

A. J. Moshayedi, Y. Xie, M. Sharifdoust, and A. S. Khan, "Evaluating OMNI robot navigation with SLAM in CoppeliaSim: Hemangiomas and nonhomogeneous paths," J. Robot. Res. (JRR), vol. 1, no. 1, pp. 7–14;

10.1109/ACCESS.2024.3355278;

doi:10.5829/ije.2025.38.07a.07.

Response: We sincerely thank the reviewers for their suggestions and guidance on our manuscript. We have carefully reviewed the two references recommended by the reviewers, and this process has also enhanced our paper writing skills. Both references pertain to robotics: one concerns the design and development of food delivery robots, while the other addresses the application of robots in corn cultivation. These topics differ from our study's focus on medical logistics drones. After careful consideration, we have chosen not to cite them. We once again express our gratitude for the reviewer's valuable suggestions.

5. What are the main objectives of the paper?

Response: At the end of the introduction, we have added a new subsection titled “1.3 Research Objectives and Questions,” which clearly outlines the purpose and motivation of this study in bullet points. This helps guide readers in understanding the paper's central focus and core objectives. Please refer to page 6 of the manuscript for the revisions.

6. How did environmental factors such as weather conditions, wind speed, or visibility affect UAV performance and flight time during the study period?

Response: We sincerely thank the reviewers for their suggestions and guidance on our manuscript. This study excluded flight data collected during severe weather conditions such as strong winds and heavy rain, thus limiting the comprehensive assessment of drone performance in extreme environments. This constitutes one of the limitations of this research. Naturally, this area warrants greater attention in future studies.

7. What are the main technical or regulatory challenges that currently limit large-scale deployment of UAV logistics in public hospitals across China?

Response: Regulatory Framework and Standards System. Considering the unique characteristics of China's airspace management, it is necessary to explore drone operation regulations and standards tailored to medical applications. These should encompass flight safety protocols, privacy protection measures, data security standards, and liability determination mechanisms to provide policy support for the industry's healthy development. This area warrants particular attention in future research.

8. Could you elaborate on how the proposed UAV logistics framework could be integrated with existing hospital information systems to ensure seamless coordination and real-time tracking of medical materials?

Response: We sincerely thank the reviewers for their suggestions and guidance on our manuscript. One limitation of this study is the insufficient depth of information system integration. The research primarily focused on transportation time metrics without delving into the issue of deep data interaction between the drone logistics system and existing hospital information systems (such as electronic medical record systems, laboratory information systems, and hospital resource planning systems). Future research will explore this topic in greater depth.

9. Overall, while the study presents valuable insights, the paper format and presentation should be revised according to the conference template. Major revisions are recommended before acceptance.

Response:We sincerely appreciate the reviewer's affirmation of our manuscript. We have thoroughly revised the formatting of the entire manuscript according to the latest submission guidelines of PLOS ONE, including but not limited to: font size, line spacing and margins, section heading styles, figure and table caption formats, and reference list styles. This ensures the manuscript fully complies with the journal's formal publication requirements in terms of presentation.

We firmly believe that through the implementation of the above revisions, the manuscript's academic quality, logical coherence, and presentation standards have been substantially enhanced. We extend our most sincere gratitude once again for your valuable time and insightful guidance.

Thank you for your consideration of this manuscript.

Yours sincerely,

Xingbo Long, M.D.

Deyang People's Hospital, Deyang, 618000, China

Tel.: +86-18280226483

E-mail: 723766496@qq.com
