# Supplementary material for: An empirical study of drone medical logistics transportation in a multi-campus model of Chinese public hospitals: Real-world data-driven validation of timeliness and application effects
Source: PLoS One. 2026 Mar 20;21(3):e0345282. doi: 10.1371/journal.pone.0345282 (PMC13004373; doi:10.1371/journal.pone.0345282)
Supplement: Attachment — Submitted filename: Response_to_Reviewers_auresp_2.docx [file pone.0345282.s008.docx]

**Point-by-point response to editors and reviewers’ comments**

Dear Editors and Reviewers,

Thank you very much for your positive feedback and for the time and effort you have dedicated to reviewing our manuscript entitled “An empirical study of drone medical logistics transportation in a multi-campus model of Chinese public hospitals: real-world data-driven validation of timeliness and application effects” (ID:PONE-D-25-54130).

We are delighted that both reviewers have indicated that all previous comments have been addressed and that the manuscript is now considered technically sound. Below, we provide a point-by-point response to the remaining minor issues raised in your latest communication.

**Response to Journal Requirements:**

1. Regarding the recommendation to cite specific previously published works

We have carefully reviewed all publications suggested by the reviewers during the previous rounds of review. Relevant works have not already been incorporated into the revised manuscript. No additional citation requirements were specified by the editor, and we confirm that no further citations are needed at this stage.

2. Regarding the completeness and correctness of the reference list

We have thoroughly reviewed the entire reference list to ensure:

All cited works are accurate and complete.

No retracted papers are included in the reference list.

All references follow the PLOS ONE format consistently.

**Response to Additional Editor Comments:**

Editor's Comment: "Thank you for submitting the revised manuscript. Although the major concerns are addressed, some minor corrections are still required."

Our Response: Thank you for this acknowledgment. We have carefully addressed all minor corrections.

**Response to Reviewer Comments:**

**Reviewer #1:**

Comment 1: "All comments have been addressed."

Our Response: Thank you for your positive assessment and for your valuable guidance throughout the review process.

Comment 2: "The revisions are accepted, please update the manuscript as it has some typos and needs proofreading."

Our Response: Thank you for this important observation. We have conducted a thorough language proofreading of the entire manuscript to eliminate any typographical or grammatical errors. Specifically:

The manuscript has been carefully reviewed by all co-authors.

We have carefully edited to ensure the manuscript is clear, correct, and unambiguous.

All identified typos and minor language issues have been corrected. Revised portion are marked in red in the manuscript.

The language-polished version of the manuscript is now submitted. We believe it meets the high standards required for publication.

**Reviewer #2:**

Comment 1: "All comments have been addressed."

Our Response: Thank you for your confirmation and for your constructive suggestions throughout the revision process.

Comment 2 (Questions 2-6): The reviewer responded "Yes" to questions regarding technical soundness, statistical analysis, data availability, and language presentation.

Our Response: We sincerely appreciate the reviewer's positive evaluation of our work.

We thank you once again for your thorough and constructive handling of our manuscript. We believe that with these final corrections, the manuscript is now suitable for publication in PLOS ONE. Should any further adjustments be required, we remain at your disposal.

Yours sincerely,

Xingbo Long, M.D.

Deyang People's Hospital, Deyang, 618000, China

Tel.: +86-18280226483

E-mail: 723766496@qq.com
